# Refinement of an indirect immunotoxin assay of monoclonal antibodies recognising the human small cell lung cancer cluster 2 antigen.

**DOI:** 10.1038/bjc.1993.232

**Published:** 1993-06

**Authors:** E. J. Derbyshire, L. de Leij, E. J. Wawrzynczak

**Affiliations:** Section of Immunology, Institute of Cancer Research, Sutton, Surrey, UK.

## Abstract

Monoclonal antibodies (Mabs) from the Second International Workshop on Small Cell Lung Cancer (SCLC) Antigens that recognise the cluster 2 SCLC-associated antigen mediated potent and selective cytotoxic effects in an indirect assay of immunotoxin cytotoxicity. In this assay, the NCI-H69 cell line was treated with each Mab at 4 degrees C, washed to remove unbound Mab, and then incubated at 37 degrees C in the presence of a fixed concentration, 1 x 10(-8) M, of the screening agent, sheep anti-mouse IgG-ricin A chain. The use of a fixed high concentration of screening agent led to a 300-fold overestimate of the potency of a cluster 2-directed immunotoxin, MOC-31-ricin A chain. In contrast, when the concentration of the screening agent was identical to the Mab concentration, a precise match to immunotoxin potency was obtained. MOC-31-ricin A chain selectivity inhibited the incorporation of [3H]leucine by the NCI-H69, SW2 and GLC-8 SCLC cell lines by 50% at a concentration between 3 x 10(-11) M and 3 x 10(-10) M, and by the NCI-H125 lung adenocarcinoma cell line at 7 x 10(-11) M, but exerted no selective toxic effects upon human lung and non-lung tumour cell lines lacking surface expression of the cluster 2 antigen.


Br. J. Cancer (1993), 67, 1242–1247 © Macmillan Press Ltd., 1993

### Refinement of an indirect immunotoxin assay of monoclonal antibodies recognising the human small cell lung cancer cluster 2 antigen

E.J. Derbyshire[1], L. de Leij[2] & E.J. Wawrzynczak[1]

[1]*Drug Targeting Group, Section of Immunology, Institute of Cancer Research, Sutton, Surrey SM2 5NG, UK, and* [2]*Department of Clinical Immunology, University Hospital, 9713EZ Groningen, The Netherlands.*

**Summary** Monoclonal antibodies (Mabs) from the Second International Workshop on Small Cell Lung Cancer (SCLC) Antigens that recognise the cluster 2 SCLC-associated antigen mediated potent and selective cytotoxic effects in an indirect assay of immunotoxin cytotoxicity. In this assay, the NCI-H69 cell line was treated with each Mab at 4°C, washed to remove unbound Mab, and then incubated at 37°C in the presence of a fixed concentration, $1 \times 10^{-8}$ M, of the screening agent, sheep anti-mouse IgG-ricin A chain. The use of a fixed high concentration of screening agent led to a 300-fold overestimate of the potency of a cluster 2-directed immunotoxin, MOC-31-ricin A chain. In contrast, when the concentration of the screening agent was identical to the Mab concentration, a precise match to immunotoxin potency was obtained. MOC-31-ricin A chain selectivity inhibited the incorporation of [$^3$H]leucine by the NCI-H69, SW2 and GLC-8 SCLC cell lines by 50% at a concentration between $3 \times 10^{-11}$ M and $3 \times 10^{-10}$ M, and by the NCI-H125 lung adenocarcinoma cell line at $7 \times 10^{-11}$ M, but exerted no selective toxic effects upon human lung and non-lung tumour cell lines lacking surface expression of the cluster 2 antigen.

Small cell lung cancer (SCLC) is a metastatic malignancy characterised by high initial response rates to conventional chemotherapy and the consequent emergence of drug resistance (Minna *et al.*, 1989). New anti-tumour agents that have the potential to act systemically and by novel mechanisms able to circumvent drug resistance could make a significant contribution to the treatment of SCLC. We have recently begun to investigate the potential of immunotoxins made by linking ricin A chain to monoclonal antibodies (Mabs) recognising SCLC-associated antigens (Wawrzynczak *et al.*, 1990, 1991, 1992; Derbyshire *et al.*, 1992). Ricin A chain immunotoxins have been administered to cancer patients systemically and selectively intoxicate target cells by inactivating ribosomes (Wawrzynczak, 1992; Wawrzynczak & Derbyshire, 1992).

Two international workshops on SCLC antigens have defined eight clusters of Mabs which react with distinct cell surface antigens associated with SCLC (Souhami *et al.*, 1987, 1991). In a preliminary study using an indirect assay of immunotoxin cytotoxicity, we identified that cluster 2 Mabs from the Second International Workshop were generally effective in delivering ricin A chain to the cytosol (Derbyshire & Wawrzynczak, 1991). From these results, we predicted that an immunotoxin made by the direct chemical linkage of ricin A chain to a cluster 2 Mab should be selectively and potently toxic to SCLC cell lines. In this study, we have constructed a cluster 2 immunotoxin, MOC-31-ricin A chain, determined its cytotoxic properties using a panel of human tumour cell lines, and have examined the factors influencing the selectivity and accuracy of the indirect assay.

### Materials and methods

### *Monoclonal antibodies from the Second International Workshop on SCLC Antigens*

Monoclonal antibody solutions distributed as part of the Second International Workshop on SCLC Antigens were either in the form of hybridoma supernatants or as dilutions of ascites/purified Mab at similar concentration. The cluster 2 Mabs from the Workshop panel that bound to greater than 95% of cells of the NCI-H69 SCLC line included the mouse Mabs MOC-31, MOC-151, PE-35, and AUAI of the IgG1 isotype, SL2.21 of the IgG2a isotype, MOC-58 and MOC-181 of undetermined isotypes, and the rat Mab LCA2 of the IgG2a isotype (Beverley *et al.*, 1991).

### *Preparation of immunotoxins*

The mouse Mab MOC-31, of the IgG1 isotype (de Leij *et al.*, 1986), recognises the SCLC-associated cluster 2 antigen (Beverley *et al.*, 1988). The mouse Mab W3/25, also of the IgG1 isotype and recognising the rat homologue of the human CD4 antigen, was used as an irrelevant control Mab. The Mabs were linked to ricin A chain via a disulphide bond according to the procedure described by Cumber & Wawrzynczak (1992). Briefly, the purified Mab was reacted with N-succinimidyl 3-(2-pyridyldithio)propionate to introduce an average of about two 2-pyridyldisulphide groups into the Mab. The derivatised Mab was allowed to react overnight with a 2.5-fold molar excess of freshly reduced ricin A chain and the mixture was applied to a column of Sephacryl S200(HR). Protein fractions of eluate were analysed by sodium dodecyl sulphate polyacrylamide gel electrophoresis and fractions corresponding to conjugate molecules consisting predominantly of one ricin A chain molecule linked to one Mab molecule were pooled to form the final preparation of immunotoxin.

Immunotoxin screening agents for use with mouse and rat Mabs were prepared by chemically linking ricin A chain to the Fab' fragments of sheep anti-mouse IgG (SAMIgG) and sheep anti-rat Ig (SARIg) respectively (Wawrzynczak *et al.*, 1990; Derbyshire & Wawrzynczak, 1991).

### *Cell lines*

The panel of human SCLC cell lines comprised the classic GLC-8 line (Postmus *et al.*, 1988), the classic NCI-H69 line (Carney *et al.*, 1985) provided by Dr L. Kelland at the Institute of Cancer Research, Sutton, UK and the variant SW2 line provided by Dr R. Stahel at the University Hospital, Zurich, Switzerland. The lung adenocarcinoma cell lines NCI-H23 and NCI-H125 (Carney *et al.*, 1985) were provided by Dr V. Macauley at the Institute of Cancer Research, Sutton, UK. The human T-lymphoblastoid cell line CEM was obtained from the American Type Tissue Culture Collection.

Cell lines were maintained in a humidified atmosphere of 5% $CO_2$ in air at 37°C. The lines were cultured in RPMI-1640 supplemented with 10% (v/v) heat inactivated foetal calf serum, 2 mM L-glutamine, 100 IU ml$^{-1}$ penicillin, and

Received 22 October 1992; and in revised form 12 January 1993.

100 $\mu$g ml$^{-1}$ streptomycin. SCLC cells growing as aggregates in suspension and adenocarcinoma cell lines growing as monolayer cultures were disaggregated to predominantly single cells for use in experiments as described previously (Wawrzynczak *et al.*, 1990). The T-lymphoblastoid cell line grew as a suspension of single cells in tissue culture. Cell suspensions for cytotoxicity assays were prepared in medium containing leucine-free RPMI (assay medium).

*Indirect immunofluorescence analysis of Mab binding to NCI-H69 cells*

NCI-H69 cells were adjusted to a density of $1 \times 10^6$ cells ml$^{-1}$ in assay medium and mixed with an equal volume of each of the cluster 2 Mab samples from the Second International Workshop. The cells were incubated on ice for 30 min and indirect immunofluorescence and flow cytometry was performed as described previously (Derbyshire & Wawrzynczak, 1991). The relative mean fluorescence intensity (MFI) of single live NCI-H69 cells was measured.

*Cytotoxicity assays*

The indirect assay of immunotoxin cytotoxicity was performed essentially as described previously (Derbyshire & Wawrzynczak, 1991). Briefly, cell suspensions at a density of $1 \times 10^5$ cells ml$^{-1}$, which had been pretreated with Mabs and washed, were incubated for 48 h at 37°C in the presence of the screening agent at a fixed concentration of $1 \times 10^{-8}$ M or in its absence. The cultures were incubated for a further 4 h in the presence of 1 $\mu$Ci of [$^3$H]leucine, were then harvested and counted for [$^3$H]leucine incorporation.

The continuous cytotoxicity assays were performed essentially as described previously (Wawrzynczak *et al.*, 1990). Briefly, cells suspensions at a density of $1 \times 10^5$ cells ml$^{-1}$ were incubated for 48 h at 37°C in the presence of immunotoxins or other agents. In the case of monolayer cultures of the adenocarcinoma cell lines, tumour cells were plated in 24-well sterile tissue culture plates at $5 \times 10^4$ cells per well. The plates were incubated for 48 h at 37°C to allow cell adherence and then treated with test samples for 24 h. Cultures were incubated for a further 4 h or 24 h in the presence of 1 $\mu$Ci of [$^3$H]leucine. Cells were then harvested and counted for [$^3$H]leucine incorporation.

### Results

*Effects of cluster 2 Mabs in the indirect assay of immunotoxin cytotoxicity relative to cell binding*

In the indirect assay of immunotoxin cytotoxicity, NCI-H69 cells were exposed to Workshop Mabs, the cells were washed to remove unbound Mab, and were then incubated in the presence of the appropriate screening agent at a concentration, $1 \times 10^{-8}$ M, which did not significantly inhibit [$^3$H]leucine incorporation in the absence of Mab. The effects on [$^3$H]leucine incorporation when the cells were treated with cluster 2 Mabs are shown in Figure 1a. The results have been plotted against the relative MFI of cells treated with each Mab sample as determined by indirect immunofluorescence and flow cytometry.

The majority of cluster 2 Mabs, including duplicate samples of MOC-31, mediated potent cytotoxic effects in combination with the screening agent reducing [$^3$H]leucine incorporation by greater than 90%. The single exception was the Mab MOC-151 which reduced [$^3$H]leucine incorporation by less than 20%. Although all the Mabs bound to greater than 95% of NCI-H69 cells, MOC-151 bound in lesser amount suggesting a possible explanation for the weaker effect with this Mab.

In a subsequent experiment, serial dilutions of hybridoma supernatants containing the cluster 2 Mabs MOC-31 and MOC-151 were compared by the indirect immunotoxin assay and indirect immunofluorescence. At similar levels of binding to NCI-H69 cells, MOC-31 and MOC-151 mediated a similar reduction in [$^3$H]leucine incorporation in combination with the screening agent (Figure 1b). This result indicated that MOC-151 shares with the other cluster 2 Mabs the property of mediating the efficient entry of ricin A chain provided that it binds to the cells at a similar level.

*Selective cytotoxic effects of MOC-31 in the indirect assay of immunotoxin cytotoxicity*

Control experiments were performed to verify that the potent cytotoxic effects of the cluster 2 Mab MOC-31 in the indirect assay were dependent upon selective binding of the intact screening agent to the cell-bound Mab. Figure 2a demonstrates that NCI-H69 cells treated with the purified MOC-31 Mab alone suffered no reduction in [$^3$H]leucine incorporation compared with untreated cells. In comparison, a large cytotoxic effect was observed when MOC-31-treated cells were incubated in the presence of SAMIgG Fab′-ricin A chain. No significant reduction in [$^3$H]leucine incorporation was observed when NCI-H69 cells which had been exposed to MOC-31 were incubated with the alkylated SAMIgG Fab′ fragment, with unconjugated ricin A chain, with a simple

**Figure 1** The relationship between the relative amounts of cluster 2 Mabs binding to NCI-H69 cells and their ability to mediate potent cytotoxic effects in the indirect assay of immunotoxin cytotoxicity. **a**, NCI-H69 cells exposed to cluster 2 Workshop Mab samples were subjected to the indirect immunotoxin assay and indirect immunofluorescence (see 'Materials and methods'). The results of the indirect assay are expressed as the incorporation of [$^3$H]leucine as a percentage of control cultures lacking screening agent. *Points* represent mean values of triplicate determinations. **b**, NCI-H69 cells exposed to dilutions of hybridoma supernatants containing MOC-31 (●) and MOC-151 (■) were analysed by the indirect assay and indirect immunofluorescence. The incorporation of [$^3$H]leucine is given as a percentage of untreated control cultures. Each point represents the mean of triplicate determinations. The error bars denote the standard deviations from the mean unless too small to be discerned.

mixture of these two components of the screening agent, or with an irrelevant immunotoxin, W3/25-ricin A chain. This result indicated that covalent coupling of ricin A chain to the SAMIgG Fab' fragment was necessary for MOC-31 to mediate potent cytotoxic effects.

In contrast to the potent cytotoxic effects seen with MOC-31 in combination with the screening agent, the combination of an isotype-matched Mab, W3/25, and the screening agent gave only a weak toxic effect that did not differ with statistical significance from the other controls in the experiment (Figure 2b). In a further control experiment, the combination of MOC-31 and the screening agent was found to be non-toxic to the T-lymphoblastoid cell line, CEM, to which binding of MOC-31 could not be detected by indirect immunofluorescence and flow cytometry (not shown). This result confirmed that the cytotoxic effects observed with MOC-31 againt the NCI-H69 cell line in the indirect assay were dependent upon the presence of the target antigen on the cell surface.

*Factors influencing the predictive value of the indirect assay*

The effect of the washing step upon the cytotoxic activity of MOC-31 in the indirect assay was assessed. In one arm of the assay, cells were exposed to MOC-31, washed to remove unbound Mab, and incubated with the screening agent at a single concentration of $1 \times 10^{-8}$ M. In the second arm of the assay, cells were exposed to MOC-31 and then incubated with the screening agent without the washing step. Comparison of the results using the two protocols revealed that the washing step made little difference to the cytotoxic effects (Figure 3a). In each case, the $IC_{50}$ was about $1 \times 10^{-12}$ M and [$^3$H]leucine incorporation was inhibited by about 95% when cells were treated with MOC-31 at a concentration of $1 \times 10^{-9}$ M. However, at the highest concentration of MOC-31, $1 \times 10^{-8}$ M, a reduction in the cytotoxic effect was observed when the washing step was omitted.

The indirect assay predicted that a MOC-31 immunotoxin made by the direct chemical attachment of ricin A chain would be potently cytotoxic to NCI-H69 cells in tissue culture. In a cytotoxocity experiment analogous with the indirect assay, MOC-31-ricin A chain was incubated with cells, the cells were washed and then incubated in fresh medium. In this assay, the $IC_{50}$ determined was only $3 \times 10^{-10}$ M, i.e. approximately 300-fold higher than that predicted by the indirect assay (Figure 3b). Thus, the indirect assay had grossly over-estimated the cytotoxic potency of MOC-31-ricin A chain. In contrast, when the assay was performed by exposing cells to MOC-31 at different concentrations, washing and then incubating in the presence of the screening agent at concentrations precisely matched to those of MOC-31, the $IC_{50}$ determined was $3 \times 10^{-10}$ M, exactly equivalent to that of MOC-31-ricin A chain (Figure 3b).

Two other experiments were performed. Firstly, the effect

**Figure 2** Cytotoxic effects of MOC-31 and W3/25 in combination with the screening agent and other agents. NCI-H69 cells exposed to **a**, MOC-31 and **b**, W3/25 at a concentration of $1 \times 10^{-8}$M for 30 min on ice were incubated in the presence of the agents indicated at a concentration of $1 \times 10^{-8}$M for 48 h. The results are expressed as a percentage of the [$^3$H]leucine incorporated by untreated control cultures. Each point represents the mean of triplicate determinations. The errors bars denote the standard deviations from the mean values.

**Figure 3** Factors influencing the predictive accuracy of the indirect assay. **a**, NCI-H69 cells pretreated with MOC-31 at the concentrations shown were either washed (●) or not washed (○) and then incubated with the screening agent at a concentration of $1 \times 10^{-8}$M. **b**, NCI-H69 cells pretreated with MOC-31-ricin A chain at the concentrations indicated were washed and incubated in medium alone (■), or cells pretreated with MOC-31 at the concentrations indicated were incubated in the presence of the screening agent at matching concentrations (□). Each point represents the mean values of triplicate determinations. The errors bars show the standard deviations from the mean values.

of a limited exposure to screening agent was tested. MOC-31-treated cells were incubated with the screening agent on ice for 30 min, were washed and then incubated in the absence of screening agent. The effects of this treatment upon [$^{3}$H]leucine incorporation were indistinguishable to the effects of treating cells in the continuous presence of the screening agent (not shown). Secondly, the effect of binding the SAMIgG Fab' fragment to the direct A chain immunotoxin was tested. Cells which had been exposed to MOC-31-ricin A chain were incubated in the continuous presence of alkylated SAMIgG Fab' fragment at a concentration of $1 \times 10^{-8}$ M. This treatment did not enhance the cytotoxic activity of MOC-31-ricin A chain (not shown).

*Specificity of MOC-31-ricin A chain action*

The toxic effects of a continuous 48 h exposure to MOC-31-ricin A chain were tested with the NCI-H69 cell line in tissue culture in parallel with an isotype-matched control immunotoxin of irrelevant specificity, W3/25-ricin A chain, with unconjugated ricin A chain, and the unconjugated MOC-31 Mab. Figure 4a shows a representative concentration-activity curve.

MOC-31-ricin A chain was toxic to NCI-H69 cells in a concentration-dependent fashion with an $IC_{50}$ of about $3 \times 10^{-11}$ M, approximately 1,000-fold lower than the concentration of unconjugated ricin A chain required to reduce [$^{3}$H]leucine incorporation by the same amount. At a concentration of $1 \times 10^{-8}$ M, MOC-31-ricin A chain reduced [$^{3}$H]-leucine incorporation by about 95%. In contrast, the isotype-matched control immunotoxin was only weakly cytotoxic and unconjugated MOC-31 Mab had no effect at the same concentrations. The $IC_{50}$ of MOC-31-ricin A chain in this continuous assay was 10-fold lower than in the discontinuous assay shown in Figure 3b presumably reflecting a greater uptake of immunotoxin upon prolonged incubation. However, this $IC_{50}$ was still 30-fold higher than that determined by the indirect assay (Figure 3a).

Unconjugated MOC-31 at a concentration of $1 \times 10^{-7}$ M inhibited the selective cytotoxic action of MOC-31-ricin A chain whereas the isotype-matched control Mab W3/25 could not block the action of the immunotoxin (Figure 4b) indicating that the antigen binding sites of MOC-31 were responsible for binding the immunotoxin to the cell surface.

**Figure 4** Toxic effects of MOC-31-ricin A chain and other agents against the NCI-H69 cell line in tissue culture. **a**, NCI-H69 cells were incubated for 48 h in the continuous presence of MOC-31-ricin A chain (■), W3/25-ricin A chain (□), MOC-31 (○), or ricin A chain (●) at the concentrations shown, and for a further 4 h in the presence of [$^{3}$H]leucine. **b**, NCI-H69 cells were incubated for 48 h in the presence of MOC-31-ricin A chain at the concentrations shown, either alone (■) or in the presence of MOC-31 (○) or W3/25 (□) each at a concentration of $1 \times 10^{-7}$M, and then for a further 4 h in the presence of [$^{3}$H]leucine. The results are expressed as a percentage of the [$^{3}$H]leucine incorporated by untreated control cultures. Each point represents the mean values of quadruplicate **a**, or triplicate **b**, determinations. The error bars denote the standard deviations from the mean.

*Toxic effects of MOC-31-ricin A chain against a panel of human tumour cell lines*

The cytotoxic activity of MOC-31-ricin A chain was assessed against a panel of human tumour cell lines in tissue culture in parallel with W3/25-ricin A chain, unconjugated ricin A chain and ricin toxin (Table I).

Ricin was potently toxic to all the cell lines in the panel with $IC_{50}$ values which ranged between $2.9 \times 10^{-13}$ M and $4.5 \times 10^{-12}$ M. MOC-31-ricin A chain was potently toxic to the SW2, NCI-H69 and GLC-8 SCLC cell lines with $IC_{50}$ values which ranged between $2.8 \times 10^{-11}$ M and $2.3 \times 10^{-10}$ M. In contrast, W3/25-ricin A chain and unconjugated ricin A chain were only weakly toxic to the SCLC cell lines with $IC_{50}$s of greater than $1 \times 10^{-8}$ M. MOC-31-ricin A chain was also potently toxic to the NCI-H125 lung adenocarcinoma cell line, which expresses the cluster 2 antigen, with an $IC_{50}$ of $6.5 \times 10^{-11}$ M.

MOC-31-ricin A chain demonstrated no selective toxic effects against the NCI-H23 lung adrenocarcinoma cell line

**Table I** Cytotoxic effects of MOC-31-ricin A chain, W3/25-ricin A chain, ricin A chain and ricin against human tumour cell lines in tissue culture. **a**, SCLC cell lines. **b**, Other cell lines

| *Agent* | | *$IC_{50}$[a](M)* | |
|---|---|---|---|
| **(A)** | *SW2* | *NCI-H69* | *GLC-8* |
| MOC-31-ricin A chain | $2.3 \pm 1.6 \times 10^{-10}$ | $2.8 \pm 2.1 \times 10^{-11}$ | $1.6 \pm 1.1 \times 10^{-10}$ |
| W3/25-ricin A chain | $3.5 \pm 0.6 \times 10^{-8}$ | $> 1.0 \times 10^{-7}$ | $> 1.0 \times 10^{-7}$ |
| Ricin A chain | $3.2 \pm 0.2 \times 10^{-8}$ | $2.6 \pm 0.4 \times 10^{-8}$ | $5.8 \pm 2.3 \times 10^{-8}$ |
| Ricin | $2.9 \pm 1.7 \times 10^{-13}$ | $7.9 \pm 3.2 \times 10^{-13}$ | $4.5 \pm 0.1 \times 10^{-12}$ |
| **(B)** | *NCI-H23* | *NCI-H125* | *CEM* |
| MOC-31-ricin A chain | $> 1.0 \times 10^{-8}$ | $6.5 \pm 5.3 \times 10^{-11}$ | $> 1.0 \times 10^{-7}$ |
| W3/25-ricin A chain | $> 1.0 \times 10^{-8}$ | $> 1.0 \times 10^{-8}$ | $> 1.0 \times 10^{-8}$ |
| Ricin A chain | $> 1.0 \times 10^{-8}$ | $> 1.0 \times 10^{-8}$ | $> 1.0 \times 10^{-8}$ |
| Ricin | $5.1 \pm 0.9 \times 10^{-13}$ | $6.3 \pm 1.8 \times 10^{-13}$ | $1.4 \pm 0.2 \times 10^{-12}$ |

[a]The $IC_{50}$s given are the mean values and standard deviations of at least three independent experiments and are quoted in terms of A chain concentration.

to which binding of MOC-31 could not be detected by indirect immunofluorescence and flow cytometry. The antigen-negative CEM cell line was also unaffected by the MOC-31 immunotoxin indicating that the action of the immunotoxin was selective for cells expressing the target antigen.

### Discussion

In this study, we have examined the specificity and accuracy of an indirect assay of immunotoxin cytotoxicity and the cytotoxic effects of an immunotoxin prepared with the Mab MOC-31 which recognises the cluster 2 antigen associated with human SCLC. The main findings of the study were: (i) similar selective cytotoxic effects were observed in the indirect assay with cluster 2 Mabs when bound to the NCI H69 SCLC cell line in similar amounts, (ii) the indirect assay grossly over-estimated the cytotoxic potency of MOC-31-ricin A chain except when the concentration of the screening agent was precisely matched to that of the Mab, and (iii) MOC-31-ricin A chain was potently and selectively toxic to human lung carcinoma cell lines expressing the cluster 2 antigen in tissue culture.

In a screen of Mabs from the Second International Workshop on SCLC Antigens by the indirect assay of immunotoxin cytotoxicity, the Mabs that mediated the most profound cytotoxic effects against the NCI-H69 cell line were those belonging to cluster 2 (Derbyshire & Wawrzynczak, 1991). This finding actually assisted in identifying some of the Mabs finally assigned to cluster 2 (Souhami *et al.*, 1991). In the initial screen, MOC-151 was the only cluster 2 Mab that bound to greater than 95% of NCI-H69 cells yet failed to mediate potent cytotoxic effects. In the present study, MOC-151 was shown to mediate cytotoxic effects similar to those of the effective cluster 2 Mab MOC-31 when the two Mabs bound to NCI-H69 cells in similar amounts, suggesting that MOC-151 had been ineffective in the initial screen because insufficient Mab had bound to the cell surface. Since both MOC-31 and MOC-151 were present in the Workshop panel at a concentration of about $1 \times 10^{-7}$ M (Manderino, 1991), the lower binding of MOC-151 might have reflected a lower avidity of binding or simply deterioration of the sample with time.

The method by which the indirect assay was performed was dictated by the nature of the Workshop Mab samples. Firstly, it had been necessary to include a washing step to remove sodium azide and any unknown contaminants present in the samples that might have affected cell viability. In this respect, the method used in this study necessarily deviated from the protocol recommended by Till *et al.* (1988) who screened purified Mabs without including a washing step. In the present study, the washing step was found to have little effect upon the cytotoxicity of MOC-31 in combination with the screening agent except at the highest concentration of Mab tested. This effect, similar to that described by Till *et al.* (1988), may have been caused by excess unbound Mab reacting with the screening agent and preventing it from binding to the cell-bound Mab. Our results suggest that a washing step may actually be beneficial when screening Mab solutions of unknown concentrations. Secondly, it had been necessary to use a fixed concentration of the screening agent in the indirect assay because the precise concentrations of Mab within the samples were unknown. Performing the assay in this way led to a 300-fold over-estimation in the potency of the MOC-31-ricin A chain immunotoxin.

A number of explanations for the apparent discrepancy between the actual potency of the MOC-31 immunotoxin and the potency estimated by the indirect assay were considered. Firstly, repeated rounds of MOC-31 internalisation might have allowed the prolonged uptake of the screening agent when present in excess. However, this explanation appeared unlikely because washing away unbound screening agent did not diminish the cytotoxic effect observed. Secondly, simple binding of the screening agent to MOC-31 might have increased internalisation of the Mab to intracellular compartments favouring ricin A chain translocation to the cytosol. However, the cytotoxic activity of MOC-31-ricin A chain was not enhanced in the presence of Fab′ fragment suggesting that binding of the Fab′ *per se* was unlikely to have altered the route of Mab entry into the cells.

Finally, we considered the possibility that the binding of multiple screening agent molecules to each molecule of MOC-31 led to a higher cytotoxic efficacy in the indirect assay. In support of this explanation, we found that the indirect assay did accurately measure the cytotoxic potency of MOC-31-ricin A chain when the concentration of the screening agent was precisely matched to that of MOC-31 thus mirroring the proportions of A chain and Mab in the MOC-31-ricin A chain immunotoxin. In the presence of excess screening agent, delivery of multiple Fab′-A chain molecules by each molecule of Mab could result in a higher delivery of ricin A chain into cells. Alternatively, even though multiple Fab′ fragments binding to MOC-31-ricin A chain did not enhance immunotoxin cytotoxicity, the multiple molecules of ricin A chain internalised with MOC-31 in the indirect assay may have altered the intracellular fate of the Mab and enhanced the process of intoxication.

In the case of the cluster 2 Mab MOC-31, the $IC_{50}$ determined by the indirect immunotoxin assay was strongly influenced by the concentration of screening agent used. The accuracy of the indirect assay also appears to depend on the target antigen recognised by the Mab because, in a previous study, we identified that the indirect assay did in fact accurately predict the cytotoxic potency of a ricin A chain immunotoxin recognising a different SCLC-associated antigen (Wawrzynczak *et al.*, 1990). Till *et al.* (1988) also reported a variability in the predictive accuracy of their indirect assay. In most cases, the cytotoxic effects of the indirect assay were closely similar to those of the ricin A chain immunotoxins but the activity of one immunotoxin was under-estimated by more than 30-fold. It would therefore seem imprudent to rely on the indirect assay to accurately predict the absolute potency of ricin A chain immunotoxins recognising different antigens.

Although MOC-31-ricin A chain was not as potently toxic as predicted by the indirect assay, the immunotoxin was shown to be selectively toxic to all three SCLC cell lines examined upon continuous exposure for 48 h. In addition to the three SCLC cell lines, MOC-31-ricin A chain was toxic to the NCI-H125 lung adrenocarcinoma cell line which expresses the target antigen. The cluster 2 antigen is an epithelial cell-surface glycoprotein with a molecular mass of about 35 kDa (Perez & Walker, 1989; Strnad *et al.*, 1989; Durbin *et al.*, 1990). This antigen has a similar predicted amino acid sequence to other cloned antigens (Linnenbach *et al.*, 1989; Simon *et al.*, 1990). These data unite the cluster 2 Mabs identified in the Workshop with other Mabs that appear to recognise the same or closely related antigens.

Two cluster 2-related Mabs, namely 17-1A and LD1, have previously been chemically linked to ricin A chain (Gilliland *et al.*, 1980; Blakey *et al.*, 1993). The immunotoxins were found to be selectively toxic to human colorectal carcinoma cells in tissue culture, with $IC_{50}$s of $1-3 \times 10^{-10}$ M, but their activities against lung carcinomas have not been reported. Immunoconjugates of cluster 2-related Mabs and chemotherapeutic drugs have been administered systemically to patients with a variety of adenocarcinomas. However, other studies have indicated that the target antigen is present and accessible to intravenously administered Mab on normal gastric mucosa *in vivo* (Shen *et al.*, 1984; Elias *et al.*, 1990). Whether this normal tissue reactivity would prove a serious limitation of immunotoxin therapy remains to be determined.

In conclusion, the cytotoxic effects measured by the indirect immunotoxin assay are sensitive to the nature of the target antigen, to the amount of Mab that binds to the target cell surface and to the relative molar amounts of Mab and screening agent. The results of indirect screens with Mabs which recognise different antigens cannot be relied upon to give a accurate guide to the relative potency of the corresponding directly-linked A chain ITs unless the Mab concentrations are defined and the concentration of the screening agent is precisely matched to that of the Mab.

This work was supported by the Cancer Research Campaign, London UK.

### References

BEVERLEY, P.C.L., OLABIRAN, Y., LEDERMANN, J.A., BOBROW, L.G. & SOUHAMI, R.L. (1991). Results of the central data analysis. *Br. J. Cancer*, **63**, Suppl. XIV, 10-19.

BEVERLEY, P.C.L., SOUHAMI, R.L. & BOBROW, L. (1988). Results of the central data analysis. *Lung Cancer*, **4**, 15-36.

BLAKEY, D.C., WRIGHT, A.F., HEWETT, P.W. & ROSE, M.S. (1993). Cytotoxicity and pharmacokinetics of a recombinant ricin A chain immunotoxin made with the monoclonal antibody LD1 recognising a human colorectal cancer antigen. In *Monoclonal Antibodies: Application in Clinical Oncology*, Epenetos, A.A. (ed.), Chapman & Hall: London (in press).

CARNEY, D.N., GAZDAR, A.F., BEPLER, G., GUCCION, J.G., MARANGOS, P.J., MOODY, T.W., ZWEIG, M.H. & MINNA, J.D. (1985). Establishment and identification of small cell lung cancer cell lines having classic and variant features. *Cancer Res.*, **45**, 2913-2923.

CUMBER, A.J. & WAWRZYNCZAK, E.J. (1992). Preparation of cytotoxic antibody-toxin conjugates. In *Methods in Molecular Biology, Vol. 10: Immunochemical Protocols*, Manson, M.M. (ed.), p. 283-293, Humana: NJ.

DE LEIJ, L., POSTMUS, P.E., POPPEMA, S., ELEMA, J.D. & THE, T.H. (1986). The use of monoclonal antibodies for the pathological diagnosis of lung cancer. In *Lung Cancer: Basic and Clinical Aspects of Lung Cancer*, Hansen, H.H. (ed.), p.31-48, Martinus Nijhoff: Amsterdam.

DERBYSHIRE, E.J., HENRY, R.V., STAHEL, R.A. & WAWRZYNCZAK, E.J. (1992). Potent cytotoxic action of the immunotoxin SWA11-ricin A chain against human small cell lung cancer cell lines. *Br. J. Cancer*, **66**, 444-451.

DERBYSHIRE, E.J. & WAWRZYNCZAK, E.J. (1991). Monoclonal antibodies recognising the cluster 2 antigen associated with human small cell lung cancer mediate the toxic effects of ricin A chain in an indirect assay of immunotoxin cytotoxocity. *Br. J. Cancer*, **63**, Suppl. XIV, 74-77.

DURBIN, H., RODRIGUES, N. & BODMER, W.F. (1990). Further characterization, isolation and identification of the epithelial cell surface antigen defined by monoclonal antibody AUA1. *Int. J. Cancer*, **45**, 562-565.

ELIAS, D.J., HIRSCHOWITZ, L., KLINE, L.E., KROENER, J.F., DILLMAN, R.O., WALKER, L.E., ROBB, J.A. & TIMMS, R.M. (1990). Phase I clinical comparative study of monoclonal antibody KS1/4 and KS1/4-methotrexate immunoconjugate in patients with non-small cell lung carcinoma. *Cancer Res.*, **50**, 4154-4159.

GILLILAND, D.G., STEPLEWSKI, Z., COLLIER, R.J., MITCHELL, K.F., CHANG, T.H. & KOPROWSKI, H. (1980). Antibody-directed cytotoxic agents: use of monoclonal antibodies to direct the action of toxin A chains to colorectal carcinoma cells. *Proc. Natl Acad. Sci. USA*, **77**, 4539-4543.

LINNENBACH, A.J., WOJCIEROWSKI, J., WU, S., PYRC, J.J., ROSS, A.H., DIETZSCHOLD, B. SPEICHER, D. & KOPROWSKI, H. (1989). Sequence investigation of the major gastrointestinal tumor-associated antigen gene family, GA733. *Proc. Natl Acad. Sci. USA*, **86**, 27-31.

MANDERINO, G.L. (1991). Measurement of murine IgG and IgM concentrations in the SCLC MAb panel; effects of concentration on sensitivity and specificity. *Br. J. Cancer*, **63**, Suppl. XIV, 64-66.

MINNA, J.D., PASS, H., GLATSTEIN, E.J. & IHDE, D. (1989). Cancer of the lung. In *Cancer: Principles and Practice of Oncology*, DeVita, V.T., Hellman, S.A. & Rosenberg, S.A. (eds.), p.591-705, Lippincott: Philadelphia.

PEREZ, M.S. & WALKER, L.E. (1989). Isolation and characterization of a cDNA encoding the KS1/4 epithelial carcinoma marker. *J. Immunol.*, **142**, 3662-3667.

POSTMUS, P.E., DE LEIJ, L., VAN DER VEEN, A.Y., MESANDER, G., BUYS, C.H.C.M. & ELEMA, J.D. (1988). Two small cell lung cancer cell lines established from bronchoscope biopsies. *Eur. J. Clin. Oncol.*, **24**, 753-763.

SHEN, J.-W., ATKINSON, B., KOPROWSKI, H. & SEARS, H.F. (1984). Binding of murine immunoglobulin to human tissues after immunotherapy with anticolorectal carcinoma monoclonal antibody. *Int. J. Cancer*, **33**, 465-468.

SIMON, B., PODOLSKY, D.K., MOLDENHAUER, G., ISSELBACHER, K.J., GATTONI-CELLI, S. & BRAND, S.J. (1990). Epithelial glycoprotein is a member of a family of epithelial cell surface antigens homologous to nidogen a matrix adhesion molecule. *Proc. Natl Acad. Sci. USA*, **87**, 2755-2759.

SOUHAMI, R.L., BEVERLEY, P.C.L. & BOBROW, L.G. (1987). Antigens of small cell lung cancer. First International Workshop. *Lancet*, **II**, 325-326.

SOUHAMI, R.L., BEVERLEY, P.C.L., BOBROW, L.G. & LEDERMANN, J.A. (1991). Antigens of lung cancer: Results of the Second International Workshop on Lung Cancer Antigens. *J. Natl Cancer Inst.*, **83**, 609-612.

STRNAD, J., HAMILTON, A.E., BEAVERS, L.S. GAMBOA, G.C., APELGREN, L.D., TABER, L.D., SPORTSMAN, J.R., BUMOL, T.F., SHARP, J.D. & GADSKI, R.A. (1989). Molecular cloning and characterization of a human adenocarcinoma/epithelial cell surface antigen complementary DNA. *Cancer Res.*, **49**, 314-317.

TILL, M., MAY, R.D., UHR, J.W., THORPE, P.E. & VITETTA, E.S. (1988). An assay that predicts the ability of monoclonal antibodies to form potent ricin A chain-containing immunotoxins. *Cancer Res.*, **48**, 1119-1123.

WAWRZYNCZAK, E.J. (1992). Rational design of immunotoxins: current progress and future prospects. *Anti-Cancer Drug Design*, **7**, 427-441.

WAWRZYNCZAK, E.J. & DERBYSHIRE, E.J. (1992). Immunotoxins: the power and the glory. *Immunol. Today*, **13**, 381-383.

WAWRZYNCZAK, E.J., DERBYSHIRE, E.J., HENRY, R.V., PARNELL, G.D., SMITH, A., WAIBEL, R. & STAHEL, R.A. (1990). Selective cytotoxic effects of a ricin A chain immunotoxin made with the monoclonal antibody SWA11 recognising a human small cell lung cancer antigen. *Br. J. Cancer*, **62**, 410-414.

WAWRZYNCZAK, E.J., DERBYSHIRE, E.J., HENRY, R.V., PARNELL, G.D., SMITH, A., WAIBEL, R. & STAHEL, R.A. (1991). Cytotoxic activity of ricin A chain immunotoxins recognising cluster 1, w4 and 5A antigens associated with human small cell lung cancer. *Br. J. Cancer*, **63**, Suppl. XIV, 71-73.

WAWRZYNCZAK, E.J., ZANGEMEISTER-WITTKE, U., WAIBEL, R., HENRY, R.V., PARNELL, G.D., CUMBER, A.J., JONES, M. & STAHEL, R.A. (1992). Molecular and biological properties of an abrin A chain immunotoxin designed for therapy of human small cell lung cancer. *Br. J. Cancer*, **66**, 361-366.


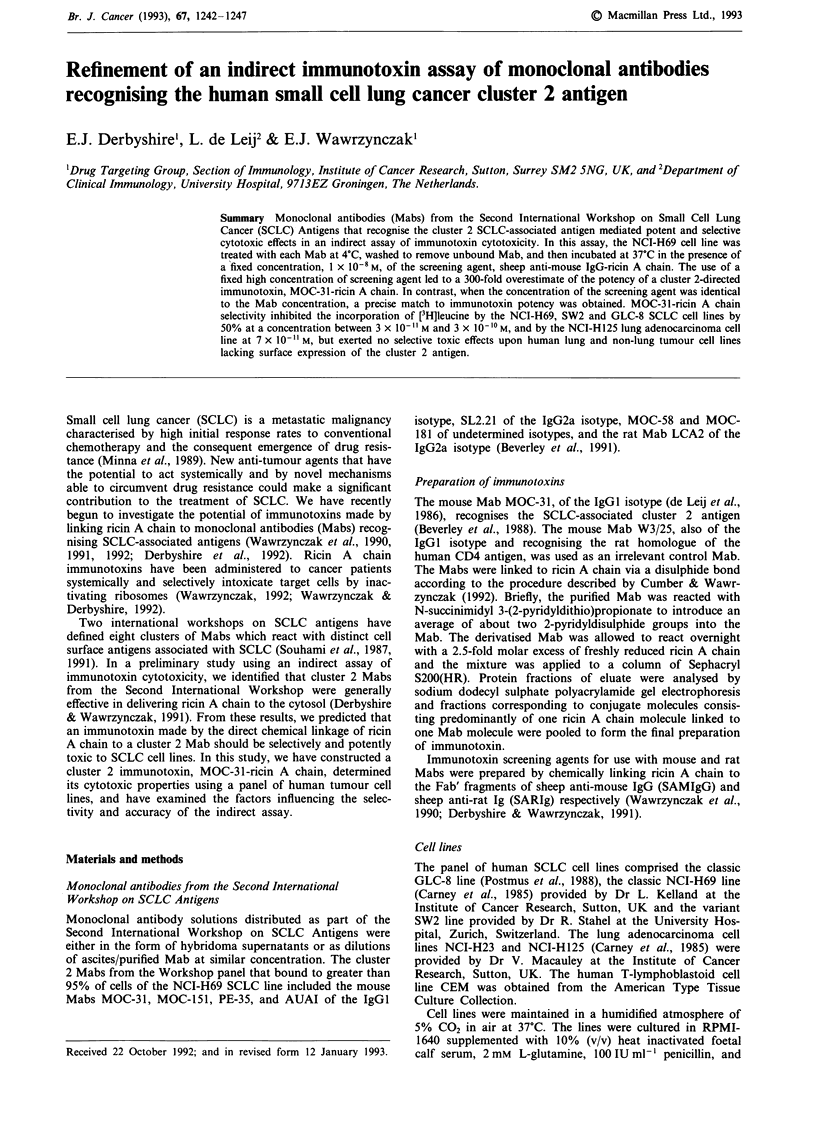

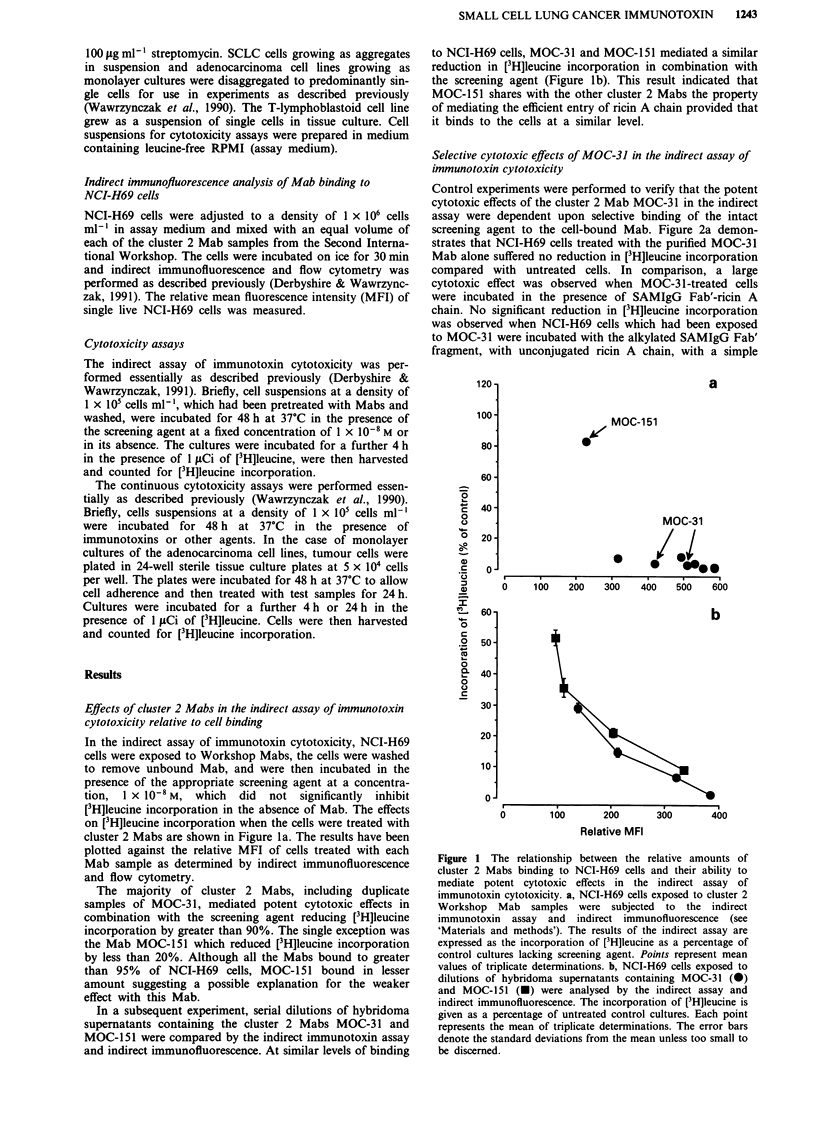

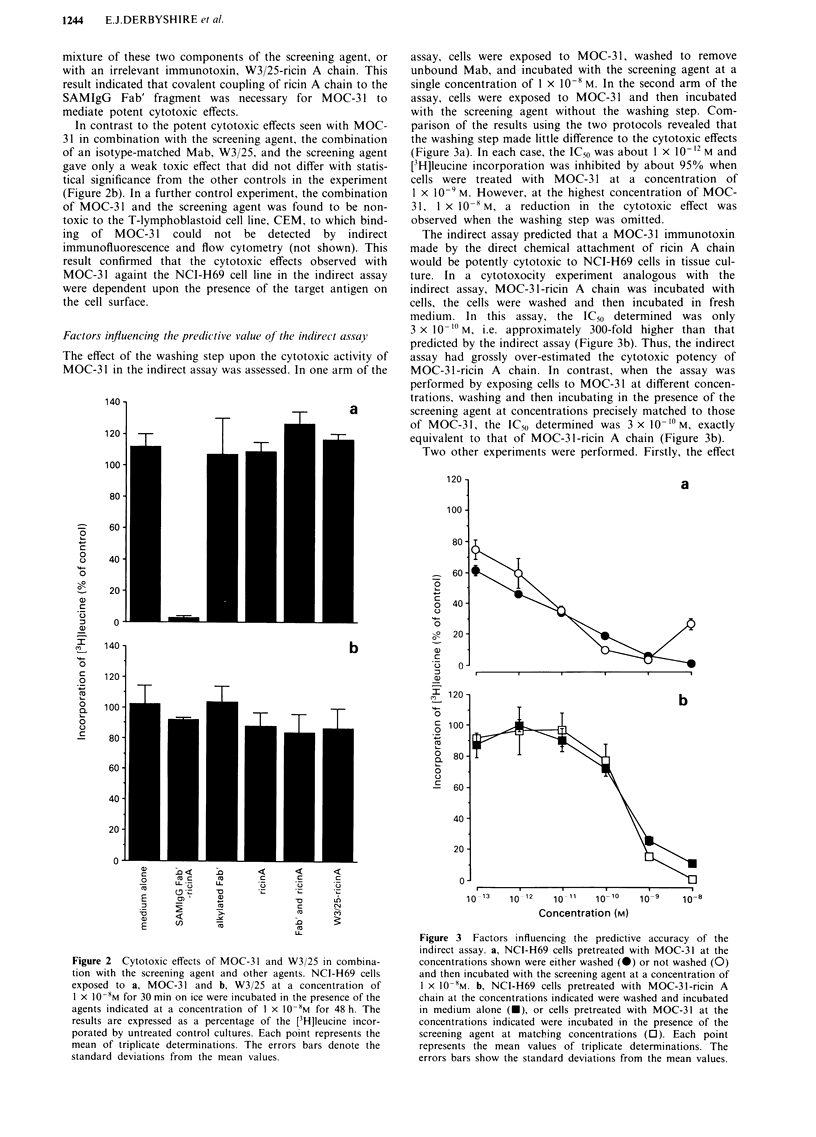

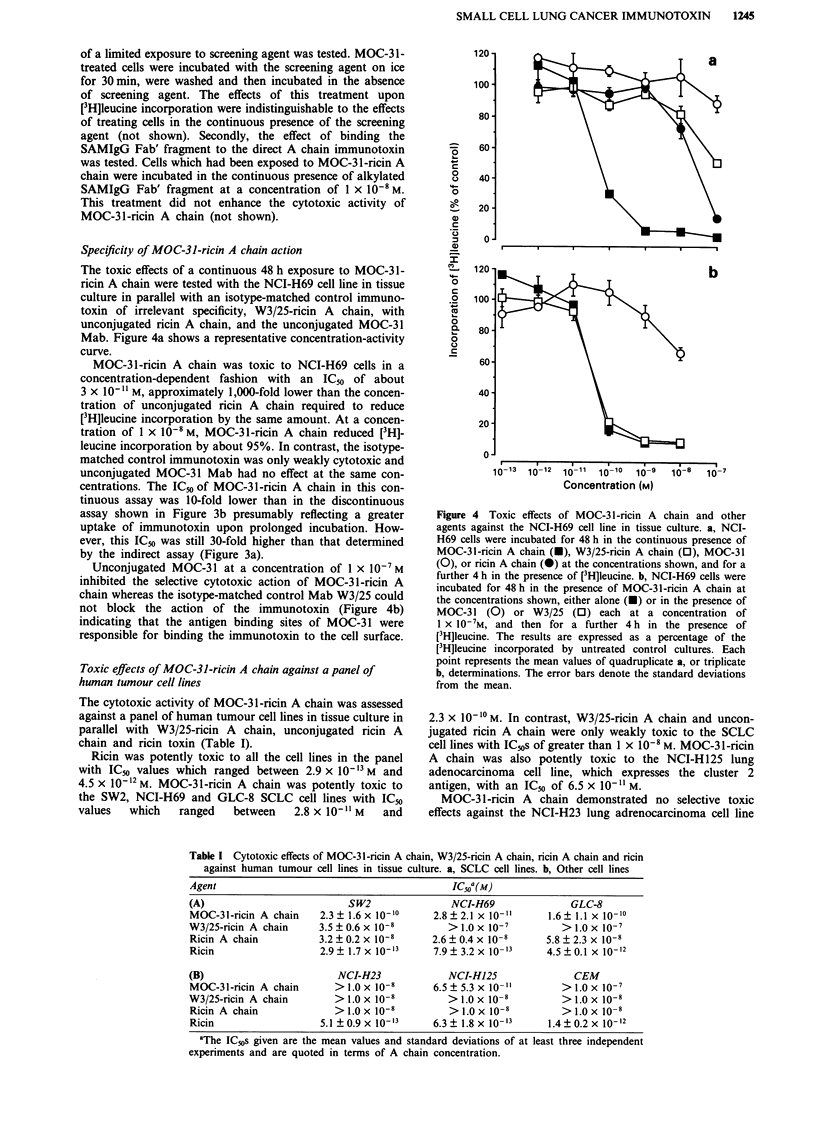

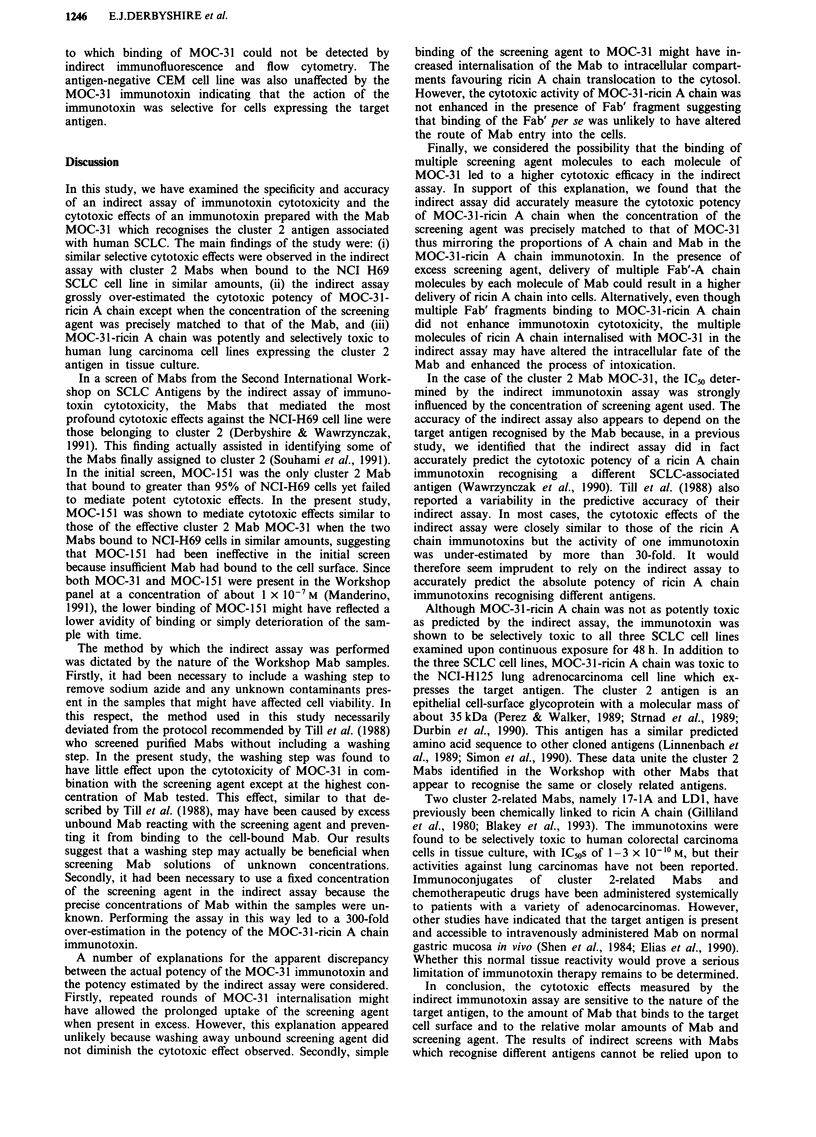

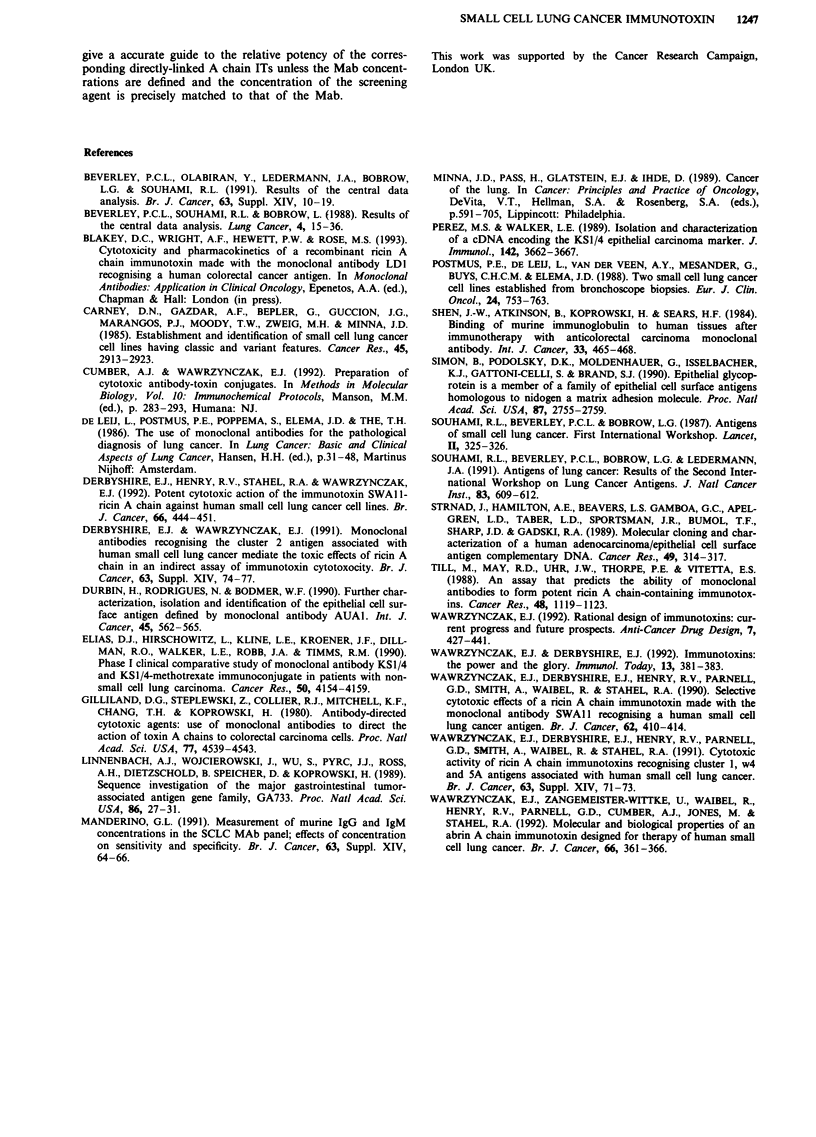


## References

[OCR_00862] Beverley P. C., Olabiran Y., Ledermann J. A., Bobrow L. G., Souhami R. L. (1991). Results of the central data analysis.. Br J Cancer Suppl.

[OCR_00879] Carney D. N., Gazdar A. F., Bepler G., Guccion J. G., Marangos P. J., Moody T. W., Zweig M. H., Minna J. D. (1985). Establishment and identification of small cell lung cancer cell lines having classic and variant features.. Cancer Res.

[OCR_00899] Derbyshire E. J., Henry R. V., Stahel R. A., Wawrzynczak E. J. (1992). Potent cytotoxic action of the immunotoxin SWA11-ricin A chain against human small cell lung cancer cell lines.. Br J Cancer.

[OCR_00905] Derbyshire E. J., Wawrzynczak E. J. (1991). Monoclonal antibodies recognising the cluster 2 antigen associated with human small cell lung cancer mediate the toxic effects of ricin A chain in an indirect assay of immunotoxin cytotoxicity.. Br J Cancer Suppl.

[OCR_00912] Durbin H., Rodrigues N., Bodmer W. F. (1990). Further characterization, isolation and identification of the epithelial cell-surface antigen defined by monoclonal antibody AUA1.. Int J Cancer.

[OCR_00920] Elias D. J., Hirschowitz L., Kline L. E., Kroener J. F., Dillman R. O., Walker L. E., Robb J. A., Timms R. M. (1990). Phase I clinical comparative study of monoclonal antibody KS1/4 and KS1/4-methotrexate immunconjugate in patients with non-small cell lung carcinoma.. Cancer Res.

[OCR_00925] Gilliland D. G., Steplewski Z., Collier R. J., Mitchell K. F., Chang T. H., Koprowski H. (1980). Antibody-directed cytotoxic agents: use of monoclonal antibody to direct the action of toxin A chains to colorectal carcinoma cells.. Proc Natl Acad Sci U S A.

[OCR_00935] Linnenbach A. J., Wojcierowski J., Wu S. A., Pyrc J. J., Ross A. H., Dietzschold B., Speicher D., Koprowski H. (1989). Sequence investigation of the major gastrointestinal tumor-associated antigen gene family, GA733.. Proc Natl Acad Sci U S A.

[OCR_00939] Manderino G. L. (1991). Measurement of murine IgG and IgM concentrations in the SCLC MAb panel; effects of concentration on sensitivity and specificity.. Br J Cancer Suppl.

[OCR_00951] Perez M. S., Walker L. E. (1989). Isolation and characterization of a cDNA encoding the KS1/4 epithelial carcinoma marker.. J Immunol.

[OCR_00956] Postmus P. E., de Ley L., van der Veen A. Y., Mesander G., Buys C. H., Elema J. D. (1988). Two small cell lung cancer cell lines established from rigid bronchoscope biopsies.. Eur J Cancer Clin Oncol.

[OCR_00962] Shen J. W., Atkinson B., Koprowski H., Sears H. F. (1984). Binding of murine immunoglobulin to human tissues after immunotherapy with anticolorectal carcinoma monoclonal antibody.. Int J Cancer.

[OCR_00968] Simon B., Podolsky D. K., Moldenhauer G., Isselbacher K. J., Gattoni-Celli S., Brand S. J. (1990). Epithelial glycoprotein is a member of a family of epithelial cell surface antigens homologous to nidogen, a matrix adhesion protein.. Proc Natl Acad Sci U S A.

[OCR_00975] Souhami R. L., Beverley P. C., Bobrow L. G. (1987). Antigens of small-cell lung cancer. First International Workshop.. Lancet.

[OCR_00980] Souhami R. L., Beverley P. C., Bobrow L. G., Ledermann J. A. (1991). Antigens of lung cancer: results of the second international workshop on lung cancer antigens.. J Natl Cancer Inst.

[OCR_00988] Strnad J., Hamilton A. E., Beavers L. S., Gamboa G. C., Apelgren L. D., Taber L. D., Sportsman J. R., Bumol T. F., Sharp J. D., Gadski R. A. (1989). Molecular cloning and characterization of a human adenocarcinoma/epithelial cell surface antigen complementary DNA.. Cancer Res.

[OCR_00993] Till M., May R. D., Uhr J. W., Thorpe P. E., Vitetta E. S. (1988). An assay that predicts the ability of monoclonal antibodies to form potent ricin A chain-containing immunotoxins.. Cancer Res.

[OCR_01015] Wawrzynczak E. J., Derbyshire E. J., Henry R. V., Parnell G. D., Smith A., Waibel R., Stahel R. A. (1991). Cytotoxic activity of ricin A chain immunotoxins recognising cluster 1, w4 and 5A antigens associated with human small cell lung cancer.. Br J Cancer Suppl.

[OCR_01008] Wawrzynczak E. J., Derbyshire E. J., Henry R. V., Parnell G. D., Smith A., Waibel R., Stahel R. A. (1990). Selective cytotoxic effects of a ricin A chain immunotoxin made with the monoclonal antibody SWA11 recognising a human small cell lung cancer antigen.. Br J Cancer.

[OCR_01004] Wawrzynczak E. J., Derbyshire E. J. (1992). Immunotoxins: the power and the glory.. Immunol Today.

[OCR_00999] Wawrzynczak E. J. (1992). Rational design of immunotoxins: current progress and future prospects.. Anticancer Drug Des.

[OCR_01022] Wawrzynczak E. J., Zangemeister-Wittke U., Waibel R., Henry R. V., Parnell G. D., Cumber A. J., Jones M., Stahel R. A. (1992). Molecular and biological properties of an abrin A chain immunotoxin designed for therapy of human small cell lung cancer.. Br J Cancer.

